# Atypical neurocognitive functioning in children and adolescents with obsessive–compulsive disorder (OCD)

**DOI:** 10.1007/s00787-023-02301-w

**Published:** 2023-11-02

**Authors:** Camilla Funch Uhre, Melanie Ritter, Jens Richardt Møllegaard Jepsen, Valdemar Funch Uhre, Nicole Nadine Lønfeldt, Anne Dorothee Müller, Kerstin Jessica Plessen, Signe Vangkilde, Robert James Blair, Anne Katrine Pagsberg

**Affiliations:** 1 The Child and Adolescent Mental Health Center, Copenhagen University Hospital – Mental Health Services CPH, Copenhagen, Denmark; 2 Center for Clinical Neuropsychology, Children and Adolescents, Rigshospitalet, Copenhagen, Denmark; 3 Department of Clinical Medicine, Faculty of Health Sciences, University of Copenhagen, Copenhagen, Denmark; 4 Center for Clinical Intervention and Neuropsychiatric Schizophrenia Research (CINS), Copenhagen University Hospital - Mental Health Services CPH, Glostrup, Denmark; 5 Center for Neuropsychiatric Schizophrenia Research (CNSR), Copenhagen University Hospital - Mental Health Services CPH, Glostrup, Denmark; 6 Danish Research Centre for Magnetic Resonance, Centre for Functional and Diagnostic Imaging and Research, Copenhagen University Hospital - Amager and Hvidovre, Copenhagen, Denmark; 7 Division of Child and Adolescent Psychiatry, Department of Psychiatry, Lausanne University Hospital and University of Lausanne, Lausanne, Switzerland; 8 Department of Psychology, University of Copenhagen, Copenhagen, Denmark

**Keywords:** Cognition, Case–control studies, Child, Adolescent, Obsessive–compulsive disorder

## Abstract

Atypical neurocognitive functioning has been found in adult patients with obsessive–compulsive disorder (OCD). However, little work has been done in children and adolescents with OCD. In this study, we investigated neurocognitive functioning in a large and representative sample of newly diagnosed children and adolescents with OCD compared to non-psychiatric controls. Children and adolescents with OCD (*n* = 119) and non-psychiatric controls (*n* = 90) underwent psychopathological assessment, intelligence testing, and a neurocognitive test battery spanning cognitive flexibility, planning and decision-making, working memory, fluency, and processing speed. The MANOVA main effect revealed that children and adolescents with OCD performed significantly worse than the control group (*p* < .001, ${\eta }_{p}^{2}$ = 0.256). Atypical patient performance was particularly found for indices of cognitive flexibility, decision-making, working memory, and processing speed. We found no evidence of differences in planning or fluency. Moreover, we found no significant associations between neurocognitive performance and OCD symptom severity or comorbidity status. Our results indicate that children and adolescents with OCD show selective atypical neurocognitive functioning. These difficulties do not appear to drive their OCD symptoms. However, they may contribute to lifespan difficulties and interfere with treatment efficacy, an objective of our research currently.

**Supplementary Information:**

The online version contains supplementary material available at 10.1007/s00787-023-02301-w.

## Introduction

Obsessive–compulsive disorder (OCD) is a common psychiatric disorder, affecting 1–3% of children and adolescents worldwide [1–3]. Core symptoms include intrusive and distressing thoughts, urges, or mental images (i.e., obsessions) and repetitive behavioral or mental rituals (i.e., compulsions) [4]. However, determining specific neurocognitive deficits in individuals with OCD, particularly in children and adolescents, has proven difficult.

An extensive body of data indicates that adult patients with OCD present with neurocognitive deficits in response inhibition, flexibility, planning, working memory (WM), and error monitoring [5]. However, meta-analyses of these data report significant deficits with small-to-medium effect sizes in the domains of processing speed, non-verbal memory, and executive functions [6–8]. Moreover, considerable heterogeneity in results and inconsistency across studies makes it difficult to draw firm conclusions [9]. Indeed, a recent umbrella review of potential biological and neurocognitive biomarkers in adults with OCD reported that the only marker showing ‘convincing’ (Class I) differential power between OCD and non-psychiatric controls were deficits in visuospatial abilities [10].

Data on children and adolescents with OCD are considerably sparser. There are indications of neurocognitive difficulties [11], but systematic reviews and meta-analyses show smaller effect sizes in children and adolescents with OCD compared to those reported in adults with OCD [12, 13]. Moreover, children and adolescents seem to show a smaller range of deficits. To our knowledge, only one meta-analysis of behavioral neurocognitive outcomes in children and adolescents with OCD has been published [12]. The meta-analysis included 11 studies covering different neurocognitive domains and found no significant group differences for children and adolescents with OCD vs. non-psychiatric controls. The direction of effects was consistent with underperformance in the OCD groups relative to non-psychiatric controls, but no differences reached statistical significance and effect sizes were small to medium, ranging from 0.04 to 0.40 across domains. A recent systematic review (without meta-analysis) of a larger number of neurocognitive studies indicated that children and adolescents with OCD show difficulties in visual WM, planning, and decision-making under uncertainty (e.g., during implicit learning of rewards) [13]—though little evidence for deficits in domains such as response inhibition and reversal learning. Given potential differences in the neurocognitive difficulties between child and adult OCD, it is critical to determine effects in pediatric samples. Some even argue that child and adult OCD should be classified as distinct psychiatric disorders given the little developmental continuity from one subtype to the other [14, 15] and the differences in symptom presentation and pattern of comorbidity [16, 17].

Several concerns emerge from the current OCD literature in children and adolescents relating to small sample sizes (though this is less of a concern recently; e.g., [11, 18–21]), comorbidities, and use of medications for OCD while participating. Medication may impact neurocognitive performance, at least in adults with OCD (see, e.g., [22]) and has been associated with changes in brain anatomy in large-scale analyses of structural neuroimaging data from the ENIGMA-OCD consortium [23].

In this study, we investigated neurocognitive functioning in a large clinical sample of children and adolescents with moderate to severe OCD compared with non-psychiatric controls. Participants had not been treated for OCD before, and their comorbidities were well characterized. Based on the prior literature, we hypothesized that OCD patients would perform worse than non-psychiatric controls on the neurocognitive tests.

## Methods

### Participants

Participants with OCD and non-psychiatric controls were recruited between September 2018 and June 2022. All participants were part of a case–control study nested in a randomized controlled trial, which included neurocognitive assessment at baseline (pre-treatment) and follow-up (post-treatment; The TECTO trial [24], clinicaltrials.gov NCT03595098). This paper presents baseline results.

All participants with OCD were recruited from psychiatric outpatient units in the Capital Region of Denmark. Inclusion criteria were age 8–17 years, primary OCD diagnosis (International Classification of Diseases, 10th revision; ICD-10) [25], and symptom severity score ≥ 16 on the Children’s Yale-Brown Obsessive–Compulsive Scale (CY-BOCS) [26]. We excluded children and adolescents with OCD if they had a full scale IQs < 70 [27, 28], had received within the past 6 months cognitive behavioral therapy (CBT), psychoeducation and relaxation therapy (PRT), antidepressants or antipsychotics, or had a diagnosis of schizophrenia, other psychotic disorder, mania, bipolar disorder, substance dependence disorder, or pervasive developmental disorder (except for Asperger’s syndrome) [24]. The TECTO trial included 130 participants with OCD. Of these, baseline neurocognitive data was collected on 119. Ninety non-psychiatric controls were recruited from the regional population via a random age-matched sample of children and adolescents, drawn from the Danish Central Person Registry [29] (for details, see Supplemental Material). Non-psychiatric controls were excluded if they had ever met the diagnostic criteria for a psychiatric disorder as per study screening or fulfilled any of the exclusion criteria also used for participants with OCD (i.e., IQ < 70).

### Procedures

Study procedures were approved by The Ethics Committee of Capital Region of Denmark (approval number: H-18010607) and The Knowledge Centre on Data Protection Compliance in The Capital Region of Denmark (VD-2018-263, I-Suite no.: 6502). Participants and their families gave informed consent prior to participation, adhering to the Declaration of Helsinki [30]. Diagnostic assessments and neurocognitive tests were administered by trained investigators (for details, see Supplemental Material). All participants received a gift card for their research activities (value ~ 38 USD per session).

### Clinical assessment

All participants were assessed for any present and/or prior psychopathology with the Kiddie Schedule for Affective Disorders and Schizophrenia—Present and Lifetime version (K-SADS-PL) [31]. OCD patients and non-psychiatric controls were screened by trained clinicians and/or master-level psychology students under supervision. OCD symptom severity in patients was assessed with CY-BOCS [26]. To evaluate presence and severity of co-occurring disorders, children and adolescents with OCD were assessed with additional tests when relevant (e.g., Autism Diagnostic Observation Schedule [32] for patients with suspected autism spectrum disorder, according to the K-SADS interview). Diagnoses were determined with a specialist in psychiatry in the outpatient clinic (for details, see Supplemental Material). Behavioral or emotional problems during testing were assessed with the Test Observation Form (TOF) [33].

### Neurocognitive tests

The test battery consisted of tests from the Cambridge Neuropsychological Test Automated Battery (CANTAB) [34], the Delis–Kaplan Executive Function System (D-KEFS) [35, 36], and the age-appropriate version of the Wechsler Scales [27, 28]. Note that we used the validated Danish versions of D-KEFS and the Wechsler Scales [36–39], while the CANTAB tasks are considered to be language independent [40]. Intelligence was assessed with the General Ability Index (GAI) from the Wechsler Scales. The remaining test battery assessed cognitive flexibility, planning and decision-making, WM, verbal and non-verbal fluency, and processing speed (Table 1). Primary outcomes from each test were chosen prior to study initiation, based on comparability with previous studies and consensus in the author group on which outcomes best reflected each function. All neurocognitive tests were administered and scored by trained psychologists and psychology master’s students under expert supervision. Standard administration and scoring procedures were followed.

**Table 1:** Neurocognitive tests and selected outcomes

Functions and tests	Test description	Outcomes
Cognitive flexibility		
Attention switching task (AST)^a^	Computerized test in which participants respond “right” or “left” based on either the direction or location of an arrow. The response rule switches continually throughout the task	Percent correct trials; switch cost^d^
Trail making test (TMT); number letter switching^b^	Paper–pencil test in which the participant draws a line connecting numbers and letters in ascending order while alternating between them (i.e., 1-A-2-B)	Completion time in seconds
Trail making test; verbal fluency switching^b^	Verbal test in which the participant names as many words as possible in 60 s, alternating between two categories (e.g., apple–table–pear–sofa)	Number of correct switches
Trail making test; design fluency, switching^b^	Paper–pencil test in which the participant draws as many different figures as possible in 60 s by connecting dots with straight lines, continually switching between filled and empty dots	Number of correct figures
Planning and decision-making		
Stockings of Cambridge (SOC)^a^	Computerized test in which the participant copies a model by rearranging items to match the model, trying to use the minimum moves required	Problems solved in min. moves^e^
Spatial working memory (SWM)^a^	Computerized test in which an increasing number of boxes are presented on the screen, and the participant is instructed to find a blue token behind one of them. When the token is found, it is hidden behind a new box and the participant is asked to avoid returning to a box where a token has already been found	Strategy^f^
Cambridge gambling task (CGT)^a^	Computerized test in which the participant guesses whether a token is hidden behind red boxes or blue boxes. On each trial, ten boxes are presented and the ratio of blue:red boxes is varied. The participant bets points on their decision, and points are won or subtracted according to outcome and bet-size	Quality of decision-making^g^; risk adjustment^h^
Working memory		
Wechsler working memory tests^c^	Visual and verbal subtests of the Wechsler Scales, including Verbal digit span, Picture span task (WISC-V) and arithmetic (WAIS-IV)	Working Memory Index (WMI) Score
Spatial working memory	*See above*	Total errors
Fluency		
Verbal fluency, phonemic^b^	Verbal test with three subtests in which the participant has 60 s to name as many words as possible beginning with a specific letter	Number of correct words
Verbal fluency, semantic^b^	Verbal test with two subtests in which the participant has 60 s to name as many words as possible from a specific semantic category	Number of correct words
Design fluency, empty dots^b^	Paper–pencil test in which the participant draws as many different figures as possible in 60 s, connecting empty dots and ignoring filled dots	Number of correct figures
Processing speed		
Trail making test; number sequencing^b^	Paper–pencil test in which the participant draws a line, connecting numbers in ascending order	Completion time in seconds
Trail making test; letter sequencing^b^	Paper–pencil test in which the participant draws a line that connects letters in alphabetical order	Completion time in seconds

^a^Tests from the CANTAB battery [33, 34].

^b^Tests from the D-KEFS battery [34–36]

^c^Tests from the Wechsler Scales: WISC-V and WAIS-IV [26–28]

^d^The difference in the average reaction time (RT) in milliseconds between switch and repeat trials

^e^The number of trials in which the participant matched the model using as few moves as possible

^f^Based on the number of times the participant started each search within a trial with a new box instead of starting with the same box and following a systematic sequence—lower scores represent more efficient strategy use

^g^The proportion of trials where the participant chose the most likely outcome—higher scores represent better decision-making

^h^The degree to which the participant adjusted the bet-size according to the ratio of red:blue boxes—higher scores means higher degree of adjustment

### Statistical analysis

Analyses were done in IBM SPSS Statistics version 25 [41].

Demographic and clinical characteristics of the groups were compared using independent-samples *t*-tests for numerical data (e.g., age) and Chi-square test for categorical data (sex). Following previous work [21], parental education in years was calculated as either the mean of both parents, or, if only one parent participated, that parent’s value was used.

Neurocognitive test performance was compared across participants with OCD and non-psychiatric controls initially, using a multivariate analysis of variance (MANOVA). This analysis was then repeated as a multivariate analysis of covariance (MANCOVA) controlling for potential effects of intelligence (as indexed via the General Ability Index; GAI). Considerable data reveal intelligence as an important determinant of neurocognitive performance [42]. Note that GAI did not include any of our neurocognitive outcome measures.

A series of exploratory analyses were conducted. First, a MANCOVA was conducted to determine an association of symptom severity (CY-BOCS total score) with test performance. Second, a group-based MANCOVA was conducted with the two processing speed measures as covariates. Third, three additional MANOVAs were conducted where the group contrasts were: (i) OCD patients with comorbidities vs. non-psychiatric controls, (ii) OCD patients without comorbidities vs. non-psychiatric controls; and (iii) OCD patients with comorbidities vs. OCD patients without comorbidities.

Effect sizes were calculated within SPSS as partial eta squared (*η*_*p*_^2^).

## Results

### Demographic and clinical characteristics

Nine patients with OCD and seven non-psychiatric controls had incomplete neurocognitive data and were thus not included in our main MANOVA analyses. The participants with OCD and the non-psychiatric control group were similar in terms of age and sex but differed significantly in GAI (intelligence), years of parental education and behavioral and emotional problems during testing as assessed with the TOF Total Score (see Table 2). Some participants with OCD were taking medications for other psychiatric/neurological conditions than OCD (Melatonin, *n* = 8 (sleep disorders); Methylphenidate, *n* = 1 (attention-deficit hyperactivity disorder); Valproate, *n* = 1 (epilepsy)).

**Table 2:** Demographic and clinical characteristics

Characteristic	OCD group Mean (*SD*), *n* (%)	Control group Mean (*SD*), *n* (%)	*p*	***η***^**2**^
Number of participants	110	83		
Number of females	57 (51.8)	42 (50.6)	0.885	
Age in years	13.44 (2.70)	13.04 (2.81)	0.321	0.005
Parental education in years	15.55 (2.09)	16.71 (1.77)	< 0.001	0.77
General Ability Index (GAI; intelligence)^a^	99.03 (12.88)	106.57 (13.53)	< 0.001	0.076
TOF^b^ Total Score	17.19 (20.20)	7.00 (7.77)	< 0.001	0.090
OCD severity				
CY-BOCS^c^ total score	25.32 (4.75)			
CY-BOCS obsessions score	12.58 (2.70)			
CY-BOCS compulsions score	12.74 (2.43)			
Co-occurring psychiatric disorders				
ADHD (F90.0)	13 (11.82)			
Asperger’s syndrome (F84.5)	13 (11.82)			
Generalized anxiety disorder (F41.1 and F93.8)	9 (8.18)			
Tourette’s disorder (F95.2)	6 (5.45)			
AD^d^ with mixed anxiety and depressed mood (F43.23)	5 (4.55)			
Other^e^	33 (30.00)			
Total with at least one co-occurring disorder	61 (55.45)			

^a^Wechsler Scales

^b^Test Observation Form

^c^Children’s Yale-Brown Obsessive–Compulsive Scale

^d^Adjustment disorder

^e^Other: F30–F39 (*n* = 1), F40–F49 (*n* = 12), F50–59 (*n* = 5), F60-69 (*n* = 1), F80-89 (*n* = 4), F90–98.9 (*n* = 13)

### Group differences in neurocognitive function

The MANOVA on group differences in neurocognitive function was highly significant for Group (F(16,176) = 3.78, *p* < 0.001, ${\eta }_{p}^{2}$= 0.256). With respect to individual neurocognitive measures, compared to non-psychiatric controls, the main effect was driven by patients with OCD showing reduced accuracy on the AST, slower RTs during TMT switch trials, poorer quality of CGT decision-making, reduced WMI, and slower RT in TMT Number-Sequencing (see Table 3). When we included the participants who were excluded from the MANOVA due to at least one missing value (*n* = 9; *n* = 7), results were comparable, and RT during TMT Letter-Sequencing turned out significant (*p* = 0.031, ${\eta }_{p}^{2}$ = 0.023; see Supplementary Material for more information).

**Table 3:** Group differences in neurocognitive test performance

Domain/task/outcome	OCD group *n* = 110		Control group *n* = 83		*p*^*a*^	*η*_*p*_^2^
	Mean	SD	Mean	SD		
Cognitive flexibility						
AST^b^ percent correct trials	91.62	8.39	93.86	5.13	0.033	0.024
AST^b^ switch cost	268.37	145.08	310.17	136.40	0.043	0.021
TMT^c^ switching, time in seconds	96.44	47.07	82.71	32.86	0.024	0.026
Verbal fluency switching^b^, correct switches	11.63	3.01	11.11	2.86	0.227	0.008
	6.96	3.10	7.25	2.75	0.487	0.003
Planning and decision-making						
SOC^b^ problems solved in minimum moves	8.10	2.16	7.64	2.93	0.209	0.008
SWM^b^ strategy	31.83	6.23	31.06	5.44	0.372	0.004
CGT^b^ quality of decision-making	0.90	0.11	0.95	0.06	< 0.001	0.070
CGT^b^ risk adjustment	1.13	0.97	1.23	0.75	0.465	0.003
Working memory						
Wechsler working memory Index^d^	98.96	12.95	106.10	12.16	< 0.001	0.073
SWM^b^ total errors	24.97	17.41	22.49	16.53	0.318	0.005
Fluency						
Verbal fluency phonemic^c^, correct words	27.70	10.12	25.81	9.32	0.185	0.009
Verbal fluency semantic^c^, correct words	38.41	10.62	37.80	8.82	0.670	0.001
	9.67	3.16	9.10	2.85	0.746	0.001
Processing speed						
TMT number-sequencing^c^, time in seconds	37.91	18.44	31.21	13.24	0.005	0.040
TMT letter-sequencing^c^, time in seconds	39.90	18.40	34.55	19.50	0.053	0.019

^a^Note *p* values reported here are from the MANOVA model, only those at *p* < 0.003 (0.05/16) should be considered significant if examined as individual tests.

^b^Tests from the CANTAB battery [33, 34]. SWM, Spatial Working Memory; AST, Attention Switching Task; SOC, Stockings of Cambridge; CGT, Cambridge Gambling Task.

^c^Tests from the D-KEFS battery [34–36]. TMT, Trail Making Task.

^d^Tests from the Wechsler Scales [26–28]

### Covariate analyses

The MANCOVA including IQ (General Ability Index; GAI) as a covariate also revealed highly significant group differences in neurocognitive function (*F*(16,169) = 3.168, *p* < 0.001, ${\eta }_{p}^{2}$ = 0.231) as well as highly significant effects on performance of intelligence (*F*(16,169) = 6.633, *p* < 0.001, ${\eta }_{p}^{2}$ = 0.386). With respect to individual neurocognitive measures, compared to non-psychiatric controls, patients with OCD showed reduced accuracy on the AST, slower RTs during TMT switch trials, poorer quality of CGT decision-making, and slower TMT number-sequencing RT (see Supplemental Table 4 for details)—note though that only the quality of CGT decision-making and working memory results would be considered significant if the tasks were examined via individual statistics.

### Exploratory analyses

The first exploratory analysis using MANCOVA with CY-BOCS total score as a covariate revealed no significant association between OCD severity and neurocognitive test performance among the patients irrespective of whether IQ were used as additional covariates or not (*F*(16,93) = 1.587, *p* = 0.088, ${\eta }_{p}^{2}$ = 0.214 and *F*(16,86) = 1.372, *p* = 0.175, ${\eta }_{p}^{2}$ = 0.203, respectively).

The second exploratory analysis examining group differences involved a group-based MANCOVA with the two processing speed measures as covariates. This again revealed highly significant group differences (*F*(14,176) = 3.615, *p* < 0.001, ${\eta }_{p}^{2}$ = 0.223) as well as highly significant associations of performance with TMT number and letter sequencing RTs (*F*(14,176) = 2.690, *p* < 0.001, ${\eta }_{p}^{2}$ = 0.176 and *F*(14,176) = 5.656, *p* < 0.001, ${\eta }_{p}^{2}$ = 0.310, respectively).

The third exploratory analysis focused on determining whether group differences were present as a function of comorbidity within the patients with OCD. The first MANOVA revealed a significant group difference for patients without comorbidities (*n* = 47) relative to typically developing children/adolescents (*F*(16,113) = 3.673, *p* < 0.001, ${\eta }_{p}^{2}$ = 0.34). The second MANOVA revealed a significant group difference for patients *with* comorbidities (*n* = 63) relative to typically developing children (*F*(16,129) = 2.784, *p* = 0.001, ${\eta }_{p}^{2}$ = 0.257). The third MANOVA revealed no significant group difference for patients with comorbidities relative to patients without comorbidities (*F*(16,93) = 0.574, *p* = 0.896, ${\eta }_{p}^{2}$ = 0.090).

## Discussion

We compared neurocognitive functioning in children and adolescents with moderate to severe OCD and non-psychiatric controls aged 8–17 years on neurocognitive measures assessing cognitive flexibility, planning and decision-making, WM, fluency, and processing speed. Analyses revealed that the patients with OCD showed markedly reduced neurocognitive performance relative to non-psychiatric controls, even after controlling for IQ as a potential covariate. These group differences were particularly driven by group performances on the AST, TMT, CGT, and WMI.

The extent to which children and adolescents with OCD show reduced neurocognitive performance relative to non-psychiatric controls has been debated with one meta-analysis reporting no significant group differences [12] while a recent review argued that they could be seen for visual WM, planning, decision-making under uncertainty and abnormal action monitoring [13]. Much of the older evidence was hampered by relatively small sample sizes. However, this has been less of a concern in recent work [11, 18–20] which, like the current study, has found evidence of group differences in neurocognitive performance.

Several important points are worth noting regarding the current results. First, the main effect of group, even for the MANCOVA including IQ and age as covariates, was highly significant. This indicates that children and adolescents with OCD do struggle with neurocognitive difficulties. Second, while groups differed in IQ—and IQ is a major determinant of neurocognitive performance (e.g., [42]), as was also seen in our data—the group difference in neurocognitive performance remained stable even after the influence of this variable was covaried out. Third, some have suggested that reduced processing speed underlies the neurocognitive underperformance seen in adults with OCD [42–44]. However, exploratory analyses with the current data indicated that the group differences in performance existed over and above group differences in processing speed. Fourth, despite the marked group differences in neurocognitive performance, our MANCOVA in the patients examining task performance and OCD symptom severity, as indexed by the CY-BOCS, revealed no significant association. In short, and consistent with previous work with adults and children/adolescents with OCD [6, 11, 45, 46], the neurocognitive difficulties indexed in the current study were not major contributors to patient symptom severity. Fifth, patients with OCD showed group differences with the non-psychiatric control participants whether these the patients presented with comorbidities or not while patients with versus without comorbidities did not differ in neurocognitive performance. These results are consistent with prior work in children/adolescents [11, 45, 46] and adults with OCD [6] and suggest that the neurocognitive difficulties identified cannot be attributed to comorbid conditions. Sixth, the neurocognitive difficulties shown by the children and adolescents with OCD were potentially particularly marked for decision-making and WM.

Difficulties in decision-making in the participants with OCD could be seen on the CGT. In line with this finding, recent studies have reported that children/adolescents with OCD, relative to comparison participants, were slower to learn response–outcome relationships and worse at adapting response strategy when previously rewarded actions were devalued compared to non-psychiatric control children [47, 48] and show poorer decision-making ability [49, 50]. The Wechsler WMI revealed group differences while the SWM index did not. Reduced working verbal and visual memory has been relatively consistently seen in adults with OCD [5] and also reported in child/adolescent samples (for a review, see [13]). Recently, reduced verbal WM was reported in one larger *N* study [11], whereas spatial WM was not in another [19].

Our study has several strengths: (i) the N was relatively large, (ii) the patients were not receiving pharmacological interventions or psychotherapy for OCD (nor had for the last 6 months) thereby avoiding potential confounding treatment effects [22, 23]; and (iii) the patient sample included a broad range of comorbidities (i.e., it was representative of real-world clinical populations). Moreover, and importantly, we could show groups of patients with and without comorbidities differed in neurocognitive performance relative to typically developing children/adolescents but did not differ from each other; i.e., the neurocognitive performance difficulties seen in the patients cannot be attributed to pathology associated with the comorbidities.

Our study also has several limitations. First, since the data presented here are cross-sectional, we were unable to evaluate if the observed neurocognitive underperformance is moderated by e.g., illness duration, earlier pharmacological or therapeutic intervention, or age of onset. Second, we cannot ensure that the patients were *naïve* to pharmacological and/or psychotherapeutic interventions for OCD as we did not assess if patients received antidepressants or antipsychotics before 6 months prior to testing. Third, we included patients with a variety of comorbidities that are known to be associated with neurocognitive underperformance. As such, it could be argued that some of our findings might relate to pathology associated with these comorbidities. Importantly, though, follow-up MANOVAs indicated that the results were not likely to be attributed to these comorbidities. Fourth, the groups differed in IQ, which is in itself a determinant of neurocognitive performance [42]. However, controlling for intelligence in secondary analyses led to results that largely mirrored those of our main analysis.

The results have clinical implications. While the atypical neurocognitive functioning observed in patients was not associated with patient symptom severity, it was highly significant and as such likely conferring some detrimental impact on patients’ lives. Moreover, this atypical neurocognitive functioning may interfere with particularly psychosocial interventions. Knowing that forms of neurocognitive dysfunction which are seen in adult patients, are already present in childhood OCD, stresses the need for studying their developmental trajectory in patients with OCD.

In conclusion, the current study indicates that many pediatric patients with OCD show a degree of atypical neurocognitive function even if the forms of atypical functioning identified here, and in previous work, do not appear to be associated with the specific symptoms associated with OCD. As such, a more precise neurocognitive model of OCD is clearly required such that it can be tested in the future with specifically designed target tests. Moreover, it will be important to determine the extent to which the neurocognitive difficulties observed here are ameliorated by treatment for OCD or, indeed, moderate treatment efficacy.

### Supplementary Information

Below is the link to the electronic supplementary material.

Supplementary file1 (DOCX 47 KB)

## Data Availability

The data presented in this article is from a longitudinal case-control study embedded in a randomized controlled trial. The trial was recently completed and main findings are not yet published. The data from the the trial and substudies are not available in a public repository at the moment.
