# Supplementary material for: Atypical neurocognitive functioning in children and adolescents with obsessive–compulsive disorder (OCD)
Source: Eur Child Adolesc Psychiatry. 2023 Nov 2;33(7):2291–300. doi: 10.1007/s00787-023-02301-w (PMC11255040; doi:10.1007/s00787-023-02301-w)
Supplement: Supplementary file 1 — Supplementary file1 (DOCX 47 KB) [file 787_2023_2301_MOESM1_ESM.docx]

**Supplementary material**

**The Danish Central Person Register (CPR**) is managed by the Danish Health Data Authority and contains personal identification numbers of all Danes, making it possible to link data across the national health data registers. It can be accessed for research by researchers. For the recruitment of non-psychiatric control children, we acquired basic information on 10 random children/adolescents for each included patient, matched on age and sex and living in the Capital Region of Denmark. We contacted parents and included children/adolescents who met inclusion criteria and consented to participate.

**Assessments** were carried out by trained investigators (including authors CFU, MR, VFU, and NNL). K-SADS and CY-BOCS were administered by medical doctors, clinical psychologists, and psychology master’s students. They trained for administering K-SADS by completing and passing an official course for clinicians at the CAMHC and for CY-BOCS by observing and getting observed by an experienced psychologist. Neurocognitive tests were administered and scored by trained psychologists and psychology master’s students under expert supervision by authors, JRMR and SV.

**The process by which diagnosis** is determined involves a clinical conference, in which a child psychiatrist or a psychologist (specialized in child psychiatry with a four-year education) evaluate all results of the psychiatric screening.

**Supplemental** **Table 1** Co-occurring disorders (*n* = 110; all co-occurring disorders included)

| **Characteristic** | **OCD group**  *n* (%) |
| --- | --- |
| Number of participants | 110 |
| Co-occurring disorders |  |
| Major Depressive Disorder, single episode, mild (F32.0) | 1 (0.91) |
| Agoraphobia (F40.0) | 1 (0.91) |
| Social Anxiety Disorder (F40.1) | 1 (0.91) |
| Specific (isolated) Phobias (F40.2) | 1 (0.91) |
| Generalized Anxiety Disorder (F41.1) | 4 (3.64) |
| Other Specified Anxiety Disorders (F41.8) | 3 (2.73) |
| Adjustment Disorder, Unspecified (F43.20) | 1 (0.91) |
| Adjustment Disorder with Depressed Mood (F43.21) | 3 (3.64) |
| Adjustment Disorder with Anxiety (F43.22) | 2 (1.82) |
| Adjustment Disorder with Mixed Anxiety and Depressed Mood (F43.23) | 5 (4.55) |
| Adjustment Disorder with Disturbance of Conduct (F43.24) | 1 (0.91) |
| Adjustment Disorder with mixed Disturbance of Emotions and Conduct (F43.25) | 1 (0.91) |
| Other Reactions to Severe Stress (F43.8) | 1 (0.91) |
| Anorexia Nervosa (F50.0) | 1 (0.91) |
| Atypical Anorexia Nervosa (F50.1) | 1 (0.91) |
| Other Eating Disorders (F50.8) | 1 (0.91) |
| Eating Disorder, Unspecified (F50.9) | 1 (0.91) |
| Other sleep disorders not due to a substance or known physiological condition (F51.8) | 1 (0.91) |
| Anxious [Avoidant] Personality Disorder (F60.6) | 1 (0.91) |
| Asperger’s Syndrome (F84.5) | 13 (11.82) |
| Other Disorders of Psychological Development (F88.0) | 2 (1.82) |
| Other Disorders of Psychological Development NEC (F88.9) | 2 (1.82) |
| Attention-Deficit Hyperactivity Disorder, Predominantly Inattentive Type (F90.0) | 13 (11.82) |
| Attention-Deficit Hyperactivity Disorder, Predominantly Hyperactive Type (F90.1) | 1 (0.91) |
| Oppositional Defiant Disorder (F91.3) | 1 (0.91) |
| Other Childhood Emotional Disorders (F93.8) | 5 (4.55) |
| Transient Tic Disorder (F95.0) | 2 (1.82) |
| Chronic Motor or Vocal Tic Disorder (F95.1) | 1 (0.91) |
| Combined Motor and Vocal Tic Disorder [de la Tourette] (F95.2) | 6 (5.45) |
| Other Tic Disorders (F95.8) | 1 (0.91) |
| Tic Disorder, Unspecified (F95.9) | 2 (1.82) |
| Enuresis Not Due To A Substance or Known Psychological Condition (F98.0) | 3 (2.73) |
| Other specified behavioural and emotional disorders with onset usually occurring in childhood and adolescence (F98.8) | 2 (1.82) |
| Total with at least one co-occurring disorder | 61 (55.45) |

**Supplemental** **Table 2** Demographic and Clinical Characteristics (*N* = 209; all subjects included)

| **Characteristic** | **OCD group**  Mean (*SD*), *n* (%) | **Control group**  Mean (*SD*), *n* (%) | ***p*** | ***η*^2^** |
| --- | --- | --- | --- | --- |
| Number of participants | 119 | 90 |  |  |
| Number of females | 62 (52.10) | 47 (52.22) | .549 |  |
| Age in years | 13.34 (2.79) | 12.93 (2.79) | .300 | .005 |
| Parental education in years | 15.56 (2.08) | 16.79 (1.75) | <.001 | .087 |
| General Ability Index (WISC-V/WAIS-IV^a^) | 98.76 (12.76) | 106.51 (13.73) | <.001 | .079 |
| TOF^b^ total score | 16.86 (19.80) | 6.99 (7.85) | <.001 | .088 |
| OCD severity |  |  |  |  |
| CY-BOCS^c^ total score | 25.49 (4.94) |  |  |  |
| CY-BOCS obsessions score | 12.70 (2.75) |  |  |  |
| CY-BOCS compulsions score | 12.79 (2.54) |  |  |  |
| Co-occurring disorders |  |  |  |  |
| Attention Deficit Hyperactivity Disorder (F90.0) | 14 (11.76) |  |  |  |
| Asperger’s Syndrome (F84.5) | 14 (11.76) |  |  |  |
| Generalized Anxiety Disorder (F41.1 and F93.8) | 9 (7.56) |  |  |  |
| Tourette’s Disorder (F95.2) | 6 (5.04) |  |  |  |
| AD^d^ with Mixed Anxiety and Depressed Mood (F43.23) | 5 (4.20) |  |  |  |
| AD with Depressed Mood (43.21) | 4 (3.36) |  |  |  |
| Other | 30 (25.21) |  |  |  |
| Total with at least one co-occurring disorder | 66 (55.46) |  |  |  |

^a^Wechsler Intelligence Scale for Children Version V; Wechsler Adult Intelligence Scale Version IV

^b^Test Observation Form

^c^Children’s Yale-Brown Obsessive Compulsive Scale

^d^Adjustment Disorder

^e^Other: F30-F39 (*n* = 1), F40-F49 (*n* = 14), F50-59 (*n* = 5), F60-69 (*n* = 1), F80-89 (*n* = 5), F90-98.9 (*n* = 14)

**Supplemental Table 3** Co-occurring disorders (*n* = 119; all patients and co-occurring disorders included)

| **Characteristic** | **OCD group**  *n* (%) |
| --- | --- |
| Number of participants | 119 |
| Co-occurring disorders |  |
| Major Depressive Disorder, single episode, mild (F32.0) | 1 (0.84) |
| Agoraphobia (F40.0) | 1 (0.84) |
| Social Anxiety Disorder (F40.1) | 2 (1.68) |
| Specific (isolated) Phobias (F40.2) | 1 (0.84) |
| Generalized Anxiety Disorder (F41.1) | 4 (3.36) |
| Other Specified Anxiety Disorders (F41.8) | 3 (2.52) |
| Acute Stress Reaction (F43.0) | 1 (0.84) |
| Adjustment Disorder, Unspecified (F43.20) | 1 (0.84) |
| Adjustment Disorder with Depressed Mood (F43.21) | 4 (3.36) |
| Adjustment Disorder with Anxiety (F43.22) | 2 (1.68) |
| Adjustment Disorder with Mixed Anxiety and Depressed Mood (F43.23) | 5 (4.20) |
| Adjustment Disorder with Disturbance of Conduct (F43.24) | 1 (0.84) |
| Adjustment Disorder with mixed Disturbance of Emotions and Conduct (F43.25) | 1 (0.84) |
| Other Reactions to Severe Stress (F43.8) | 1 (0.84) |
| Anorexia Nervosa (F50.0) | 1 (0.84) |
| Atypical Anorexia Nervosa (F50.1) | 1 (0.84) |
| Other Eating Disorders (F50.8) | 1 (0.84) |
| Eating Disorder, Unspecified (F50.9) | 1 (0.84) |
| Other sleep disorders not due to a substance or known physiological condition (F51.8) | 1 (0.84) |
| Anxious [Avoidant] Personality Disorder (F60.6) | 1 (0.84) |
| Other Developmental Disorders of Scholastic Skills (F81.8) | 1 (0.84) |
| Asperger’s Syndrome (F84.5) | 14 (11.76) |
| Other Disorders of Psychological Development (F88.0) | 2 (1.68) |
| Other Disorders of Psychological Development NEC (F88.9) | 2 (1.68) |
| Attention-Deficit Hyperactivity Disorder, Predominantly Inattentive Type (F90.0) | 14 (11.76) |
| Attention-Deficit Hyperactivity Disorder, Predominantly Hyperactive Type (F90.1) | 1 (0.84) |
| Oppositional Defiant Disorder (F91.3) | 1 (0.84) |
| Phobic Anxiety Disorder of Childhood (F93.1) | 1 (0.84) |
| Other Childhood Emotional Disorders (F93.8) | 5 (4.20) |
| Transient Tic Disorder (F95.0) | 2 (1.68) |
| Chronic Motor or Vocal Tic Disorder (F95.1) | 1 (0.84) |
| Combined Motor and Vocal Tic Disorder [de la Tourette] (F95.2) | 6 (5.04) |
| Other Tic Disorders (F95.8) | 1 (0.84) |
| Tic Disorder, Unspecified (F95.9) | 2 (1.68) |
| Enuresis Not Due To A Substance or Known Psychological Condition (F98.0) | 3 (2.52) |
| Other specified behavioural and emotional disorders with onset usually occurring in childhood and adolescence (F98.8) | 2 (1.68) |
| Total with at least one co-occurring disorder | 66 (55.46) |

**Supplemental** **Table 4** Group Differences in Neurocognitive Test Performance (*N* = 209; all subjects included)

|  | **OCD Group**  *n* = 119 | | **Control Group**  *n* = 90 | |  |  |
| --- | --- | --- | --- | --- | --- | --- |
| **Domain/task/outcome** | **Mean** | **SD** | **Mean** | **SD** | ***p^a^*** | ***η_p_*^2^** |
| Cognitive Flexibility |  |  |  |  |  |  |
| AST^b^ Percent Correct Trials | 91.58 | 8.28 | 93.80 | 5.10 | .027 | .024 |
| AST^b^ Switch Cost | 267.83 | 142.46 | 309.77 | 134.66 | .034 | .022 |
| TMT^c^ Switching, Time in Seconds | 96.34 | 46.70 | 82.83 | 32.08 | .020 | .026 |
| Verbal Fluency Switching^c^, Correct Switches | 11.47 | 3.03 | 11.24 | 2.88 | .569 | .002 |
| Design Fluency Switching^c^, Correct Figures | 6.85 | 3.13 | 7.24 | 2.77 | .350 | .004 |
| Planning and Decision-making |  |  |  |  |  |  |
| SOC^b^ Problems Solved in Minimum Moves | 8.04 | 2.14 | 7.59 | 2.99 | .205 | .008 |
| SWM^b^ Strategy | 31.84 | 6.06 | 31.08 | 5.31 | .347 | .004 |
| CGT^b^ Quality of Decision Making | .89 | .11 | .95 | .06 | <.001 | .080 |
| CGT^b^ Risk Adjustment | 1.12 | .96 | 1.13 | .79 | .251 | .007 |
| Working Memory |  |  |  |  |  |  |
| Wechsler Working Memory Index^d^ | 98.45 | 13.07 | 105.93 | 12.22 | <.001 | .079 |
| SWM^b^ Total Errors | 25.15 | 17.06 | 22.39 | 16.85 | .246 | .007 |
| Fluency |  |  |  |  |  |  |
| Verbal Fluency Phonemic^c^, Correct Words | 27.31 | 10.21 | 25.52 | 9.43 | .202 | .008 |
| Verbal Fluency Semantic^c^, Correct Words | 37.94 | 10.57 | 37.51 | 8.85 | .755 | .000 |
| Design Fluency^c^, Correct Figures | 9.59 | 3.15 | 9.59 | 2.90 | .995 | .000 |
| Processing Speed |  |  |  |  |  |  |
| TMT Number-Sequencing^c^, Time in Seconds | 38.25 | 18.29 | 31.08 | 12.91 | .002 | .047 |
| TMT Letter-Sequencing^c^, Time in Seconds | 40.09 | 18.28 | 34.42 | 18.85 | .031 | .023 |

^a^Note only *p*-values at *p* < .003 (0.05/16) survive multiple comparison correction

^b^Tests from the CANTAB battery [33]. SWM, Spatial Working Memory; AST, Attention Switching Task; SOC, Stockings of Cambridge; CGT, Cambridge Gambling Task

^c^Tests from the D-KEFS battery [34, 35]. TMT, Trail Making Task

^d^Tests from the Wechsler Scales [26, 27]

**Supplemental Table 3** Group Differences in Neurocognitive Test Performance **(**Covariate analyses; IQ)

|  | **OCD Group**  *n* = 104 | | **Control Group**  *n* = 83 | |  |  |
| --- | --- | --- | --- | --- | --- | --- |
| **Domain/task/outcome** | **Mean** | **SEM** | **Mean** | **SEM** | ***p^a^*** | ***η_p_*^2^** |
| Cognitive Flexibility |  |  |  |  |  |  |
| AST^b^ Percent Correct Trials | 91.85 | .61 | 93.54 | .68 | .129 | .012 |
| AST^b^ Switch Cost | 279.85 | 13.47 | 304.71 | 15.14 | .248 | .007 |
| TMT^c^ Switching, Time in Seconds | 94.41 | 3.18 | 85.10 | 3.58 | .137 | .012 |
| Verbal Fluency Switching^c^, Correct Switches | 11.74 | .24 | 10.93 | .27 | .073 | .017 |
| Design Fluency Switching^c^, Correct Figures | 6.95 | .23 | 7.14 | 0.26 | .669 | .001 |
| Planning and Decision-making |  |  |  |  |  |  |
| SOC^b^ Problems Solved in Minimum Moves | 8.18 | .24 | 7.48 | .27 | .069 | .018 |
| SWM^b^ Strategy | 31.76 | .55 | 31.24 | .62 | .563 | .002 |
| CGT^b^ Quality of Decision Making | .90 | .01 | .94 | .01 | <.002 | .050 |
| CGT^b^ Risk Adjustment | 1.20 | .08 | 1.18 | .09 | .888 | .000 |
| Working Memory |  |  |  |  |  |  |
| Wechsler Working Memory Index^d^ | 101.09 | 1.06 | 103.95 | 1.19 | .079 | .017 |
| SWM^b^ Total Errors | 24.43 | 1.50 | 23.49 | 1.69 | .704 | .001 |
| Fluency |  |  |  |  |  |  |
| Verbal Fluency Phonemic^c^, Correct Words | 28.27 | .77 | 25.05 | .87 | .029 | .026 |
| Verbal Fluency Semantic^c^, Correct Words | 38.74 | .81 | 37.09 | .91 | .260 | .007 |
| Design Fluency^c^, Correct Figures | 9.76 | .26 | 9.37 | .29 | .393 | .004 |
| Processing Speed |  |  |  |  |  |  |
| TMT Number-Sequencing^c^, Time in Seconds | 38.45 | 1.44 | 31.51 | 1.62 | .017 | .031 |
| TMT Letter-Sequencing^c^, Time in Seconds | 39.90 | 1.56 | 35.90 | 1.76 | .362 | .005 |

^a^Note *p*-values reported here are from the MANOVA model, only those at *p* < .003 (0.05/16) should be considered significant if examined as individual tests.

^b^Tests from the CANTAB battery [33]. SWM, Spatial Working Memory; AST, Attention Switching Task; SOC, Stockings of Cambridge; CGT, Cambridge Gambling Task

^c^Tests from the D-KEFS battery [34, 35]. TMT, Trail Making Task

^d^Tests from the Wechsler Scales [26, 27]
